# Carcinome basocellulaire tatoué

**DOI:** 10.11604/pamj.2013.14.113.2603

**Published:** 2013-03-23

**Authors:** Sanaa Lemtibbet, Badreddine Hassam

**Affiliations:** 1 Service de Dermatologie. CHU Ibn Sina. Rabat, Maroc

**Keywords:** Carcinome basocellulaire tatoué, tumeur épithéliale, tissu épidermique, exérèse chirurgicale, Basal cell carcinoma, tattooed, epithelial tumor, epidermal tissue, surgical excision

## Image en médicine

Le carcinome basocellulaire est une tumeur épithéliale développée aux dépens du tissu épidermique, survenant le plus souvent de novo, localisée uniquement à la peau, et de malignité locale. Son pronostic est relativement favorable mais un traitement précoce, essentiellement chirurgical est nécessaire vu son potentiel important de destruction tissulaire à l'origine d'une forte morbidité. De nombreuses formes cliniques et histologiques sont décrites, à savoir le carcinome basocellulaire tatoué ou pigmenté qui pose le problème de diagnostic différentiel avec le mélanome. Nous rapportons le cas d'un patient âgé de 70 ans de phototype IV qui présentait depuis 5 ans une lésion nodulaire du vertex augmentant progressivement de taille et devenant saignante au moindre contact. L'examen objectivait une tumeur ulcèro- bourgeonnante, grossièrement arrondie, de 4cm de grand axe, de couleur chair reposant sur un plaque pigmentée noirâtre et à bordure perlée. La biopsie de la tumeur objectivait un foyer tumoral constitué de cellules basaloïdes avec noyaux agencés en palissade en périphérie et présence d'artefacts de rétraction confirmant le diagnostic de carcinome basocellulaire. Le patient a bénéficié d'une exérèse chirurgicale avec des marges de sécurité de 4 mm. L'examen histologique de la pièce opératoire montrait que les marges étaient saines. Une cicatrisation dirigée était préconisée après le geste avec réépidémisation complète après un mois.

**Figure 1:**
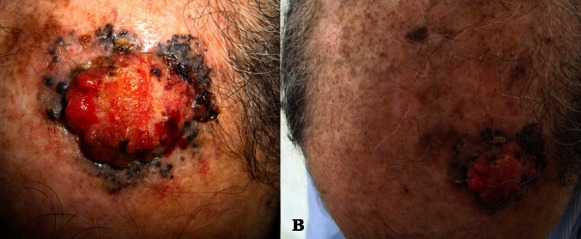
A: Tumeur ulcèro- bourgeonnante, grossièrement arrondie, de 4cm de grand axe, de couleur chair reposant sur un plaque pigmentée noirâtre et à bordure perlée. B: Multiples kératoses séborrhéiques et actiniques du scalp

